# Morphology, Rheology and Crystallization in Relation to the Viscosity Ratio of Polystyrene/Polypropylene Polymer Blends

**DOI:** 10.3390/ma13040926

**Published:** 2020-02-19

**Authors:** Salim Hammani, Nadji Moulai-Mostefa, Pieter Samyn, Mikhael Bechelany, Alain Dufresne, Ahmed Barhoum

**Affiliations:** 1 Laboratoire de Chimie Physique Moléculaire et Macromléculaire, Faculté de Science, Université de Blida, 109000 Blida, Algeria; hammani71@yahoo.fr; 2 Laboratoire Matériaux et Environnement, Faculté des Sciences & Technologie, Université de Médéa, Ain D’Heb, 26001 Medea, Algeria; moulai_nadji@yahoo.fr; 3 Applied and Analytical Chemistry, Institute for Materials Research (IMO-IMOMEC), Hasselt University, 3590 Diepenbeek, Belgium; pieter.samyn@uhasselt.be; 4 Institut Européen des Membranes IEM, UMR 5635, Univ Montpellier, ENSCM, CNRS, 34095 Montpellier CEDEX 5, France; 5 Univ. Grenoble Alpes, CNRS, Grenoble INP, LGP2, 38000 Grenoble, France; alain.dufresne@pagora.grenoble-inp.fr; 6 Chemistry Department, Faculty of Science, Helwan University, Ain Helwan, Cairo 11795, Egypt

**Keywords:** polymer blends, morphology, crystallization, rheology

## Abstract

Microfibrillar and droplet morphology of polypropylene (PP) phase dispersed in polypropylene (PS) was fabricated by using melt-extrusion. This morphology was obtained by introducing isotactic PP (20 wt.%) with different viscosity in the PS matrix (80 wt.%). Furthermore, the rheological properties of the blend investigated as a function of the viscosity ratio *K*. The variations in blend morphology were related to crystallization, melting properties, and viscoelasticity. The blends with *K* >> 1 develop a fine morphology with PP microfibrils along the flow direction, while diameters of the dispersed PP droplets gradually increase with lower values of *K* = 1, or *K* << 1. Crystallinity of the prepared blends significantly decreases compared to neat PP, while the microfibrillar morphology induces homogeneous crystallization with small crystallites. This is reflected in a decrease of the crystallization temperature, small loss in the crystallinity, and lower melting temperature of the PS80/PP20 blend compared to neat PP. The storage moduli, loss moduli, and complex viscosity are highest for the microfibrillar morphology that presents retarded relaxation. The rheological properties are dominated by the dispersed phase (*K* > 1), or matrix (*K* < 1). The variation in blend properties with microfibrillar morphology can be clearly distinguished from heterogeneous blends containing PP droplets, providing an efficient tool to create a binary blend with unique properties.

## 1. Introduction

Polymer blending is a simple and economically viable route to develop composite materials with superior properties than the parent phases. While some properties of immiscible binary blends such as homogeneity, elongation, and impact strength improve by interface compatibilization, the tensile strength for non-compatibilized binary blends of polystyrene (PS) with polypropylene (PP) remains superior in the non-compatibilized form [1]. Therefore, non-compatibilized PS/PP blends can be favorable for packaging materials with low water vapor transmission [2]. Besides extensive research on chemical interface compatibilization [3], the PS/PP blends were less frequently studied in their non-compatibilized form [4]. No chemical interactions and poor adhesion between the phases should be expected, but the internal stresses may contribute to partial compatibility and physical interactions in PS/PP blends with PS fractions up to 30 wt.% [5]. The higher percentages of PS up to 70 wt.% may induce phase inversion from continuity to co-continuity within a polyolefin blend, with a shift of the percolation threshold for dispersed PS to higher concentrations [6]. However, general relationships between blend composition and properties cannot be drawn due to contradictory results and lacking information on intermediate relationships between composition, processing, and morphology. The creation of specific morphologies as a function of the composition is a key factor, where the refinement of the dispersed phase improves mechanical properties and/or provides mechanical dampening properties through energy dissipation at the interface between the matrix phase (i.e., predominant or continuous phase) and dispersed phase (i.e., discontinuous phase) [7]. The formation of finely dispersed particles frequently occurs in ternary blends as a result of wetting effects, but it is more difficult to induce a fine morphology in immiscible binary blends that present a rather island-like phase organization [8] or a bimodal distribution [9].

The morphology of the dispersed phase in binary blends generally occurs as droplets under normal operation, or filaments with large form factor under controlled conditions [10,11,12,13]. The size, shape, and spatial distribution of both phases result from a complex correlation between composition (molecular weight and polarity), properties (viscosity, interfacial adhesion, and elasticity), and processing conditions (mixing design and temperature, time) [14,15]. The morphology depends on the dynamic effects of break-up and coalescence of dispersed domains under shear [16], which may specifically lead to in situ generated fibrillar structures [17]. In particular, the deformation of dispersed PS droplets was observed in immiscible PP/PS blends under uniaxial elongation flow, depending on the local temperature during melt-spinning [18]. The PP/PS blends additionally filled with silica nanoparticles are sensitive to elongational deformation and thinning of the droplets depending on the stabilization of the interfacial tension [19]. The morphology influences the thermal and rheological properties, as the elastic response directly related to the size and shape of the dispersed phase that is characterized by relaxation time [20]. The relaxation time is proportional to the ratio of viscous forces working to deform the droplet geometry in dispersing flows and the interfacial tension working to restore the droplet to its original shape, as expressed by the capillary number Ca=ηmrdγ˙Γ12, where *η_m_* is the viscosity of the matrix phase, *r_d_* is the diameter of the dispersed phase, $\dot{\gamma }$ is the shear rate, and Γ_12_ is the interfacial tension [21]. The interfacial tension for PS/PP blends was studied as a function of the molecular weight of PS and a single PP grade, and it increases with an increasing molecular weight of PS, while it levels off at large values [22]. The morphology of polymer blends and critical composition for co-continuity were mainly studied through control of interfacial tension by changing the composition and viscosity of the PS matrix [23].

The rheological properties of binary blends are characterized by the viscosity ratio =ηdηm, where *η_d_* and *η_m_* are the viscosities of the dispersed and matrix phase. The increase of viscosity ratio induces a larger diameter of the dispersed phase, forming microfibrils for polyethylene terephthalate (PET)/polypropylene (PP) blends [24]. However, the role of the viscosity ratio in fibrillar morphology has not yet been clearly demonstrated and understood for other blends, due to strong interferences with specific blend composition. For the PS/PP blends, variations in blend morphologies were studied as a function of the blend composition (i.e., PS/PP weight ratio) [5,25], where the viscosity ratio was only varied through a change in the viscosity of the PS matrix phase [26]. However, the shear viscosity of PP/PS blends deviates from linearity as a function of blend composition [27], and different modes of dispersion and sizes of the dispersed phase can induce pronounced differences in rheological properties of PP/PS blends [28]. The change in viscosity ratio of PP/PS blends through a variation in the composition of the amorphous PS matrix phase induced droplet break-up at a concentration of dispersed phase up to 20 wt.%, and low viscosity ratios offer the finest morphology [29]. Otherwise, the mean size of PS as a dispersed phase in compatibilized binary PP/PS blends decreases with increasing viscosity of the PP matrix [30], or the shape of dispersed PS particles becomes smallest at *K* < 1 [4].

The crystallization phenomena in polymer blends were studied for various compositions [31,32,33], including two semi-crystalline polymers [34,35], or a semi-crystalline with an amorphous polymer [7,36]. When the semi-crystalline phase was the matrix, the degree of crystallinity was not affected by the addition of the amorphous polymer, and the amount of semi-crystalline phase dominated the evolution of crystallinity [37,38]. Alternatively, a fractioned crystallization happened when the dispersed phase was formed by a semi-crystalline in an amorphous matrix [39]. The presence of crystallization peaks at a lower temperature was attributed to the confined areas and homogeneous nucleation induced during high supercoiling [40]. A decrease in the degree of crystallinity for PP with the incorporation of rubber was observed and attributed to incomplete crystallization [41]. In general, mechanical pinning at the interface of PP dispersed into PS influenced crystallization and relaxation temperatures [42].

Although immiscible PS/PP blends are frequently studied as a model for binary polymer blends, the PS is mostly considered as the dispersed phase and/or compositional studies were done by varying the PS grade. Contrary to major literature, in our work, the PS was considered to be the matrix with constant composition and blended with PP grades of different molecular weight (viscosity) as the dispersed phase. The blends were prepared by melt-extrusion of PS as a matrix (amorphous) and isotactic PP as a dispersed phase (semi-crystalline) in a fixed ratio (PS/PP = 80/20 wt.%), using four PP grades with different viscosities. The effects of the viscosity ratio (K) on the relationships between morphology, rheological properties, and crystallization are studied. In particular, the spontaneous occurrence of a unique microfibrillar structure provides a tool to control the blend morphology by selecting a suitable viscosity ratio.

## 2. Materials and Methods

### 2.1. Materials

The materials used in this study were commercial-grade polymers. A polystyrene (PS) grade was supplied by BASF (Ludwigshafen, Germany), with average molecular weight M_w_ = 215160 g/mol, polydispersity index M_w_/M_n_ = 2.35, and zero shear viscosity *η*_0_ = 3150 Pa·s at 200 °C (see later). Several grades of isotactic polypropylene (iPP) with different viscosities were produced by Ineos (Rolle, Switzerland) and Repsol (Madrid, Spain). The material characteristics are collected in Table 1. The polymer pellets were dried in a vacuum oven, at 80 °C, for 18 h, before further use.

### 2.2. Compounding Procedure

The PS80/PP20 blends were prepared with a fixed ratio of 80 wt.% PS/20 wt.% PP, including different viscosity grades of PP as a dispersed phase. The compounding was done by melt-extrusion, using a twin-screw mini extruder (Haake MiniLab Rheomex CTW5, Thermo Fischer, Karlsruhe, Germany) at 200 °C and 60 rpm, for 10 min, followed by air-cooling [43]. The screw profile was adapted with consecutive elements for dispersive and distributive mixing. The mixing time of 10 min was sufficient to obtain a homogeneous distribution of PP in the PS matrix at a relatively constant torque without degradation of the material. The blends were subsequently compression molded into samples with 25 mm diameter and 2 mm thickness in a hydraulic press (Carver Inc., Wabash, IN, USA) at 200 °C for 15 min and 12 tons, while the degassing of samples was done for 3 times, followed by cooling to room temperature. Prior to processing, the extruded blends were additionally vacuum-dried. The PS80/PP20 blends with composition and sample codes are summarized in Table 2, including samples with different viscosity ratio, $\eta_{0,\mathrm{PP}}/\eta_{0,\mathrm{PS}}$ = *K* >> 1, *K* ≈ 1, and *K* << 1, by varying the viscosity of the dispersed PP phase.

### 2.3. Determination of Phase Morphology

The phase morphology of the blends was examined by scanning electron microscopy (SEM, S-3340, Hitachi Ltd., Krefeld, Germany), operating at 15 kV accelerating voltage, as done for previous blends [44]. The surface of the samples observed by SEM was obtained by cryogenically fracturing in liquid nitrogen, along two orthogonal sections relative to the flow (i.e., transverse and longitudinal directions). The examined surface was coated with a gold-palladium thin film to avoid charging on the fractured surface. Both number-average diameter (*d_n_*) and weight-average diameter (*d_w_*) of the dispersed phase (PP) were determined from frequency histograms obtained by statistical image processing (Image J v.1.8.0) of SEM images, including 50 particles at independent locations. A statistical correction was applied to the diameter distribution according to the method of Saltikov for biaxial orientation [45].

### 2.4. Melting and Crystallization Behavior

The melting and crystallization behavior of virgin PP grades with different viscosities, virgin PS grade, and PS80/PP20 blends was investigated by differential scanning calorimetry (DSC, TA2920, TA Instruments, New Castle, DE, USA), in a nitrogen atmosphere [44]. A sample of approximately 15 mg was first heated rapidly from room temperature to 250 °C and held at this temperature for 5 min, in order to eliminate water traces and erase previous thermal history. The sample was cooled down to −30 °C, at a controlled rate of 10 °C/min, and maintained for 5 min, at the lowest temperature, while recording the crystallization properties. Afterward, the sample was heated in a second run to 250 °C, at the same rate of 10 °C/min, recording the melting properties. The degree of crystallinity, *X_c_*, was evaluated by the following formula (Equation (1)):

(1) $$X_{c}\;\left(\%\right)=\frac{\Delta H_{m}}{\Delta H_{m}^{{}^{\circ}}\times \varphi_{\mathrm{PP}}}\times 100\;$$

where $\Delta H_{m}$ is the value of the enthalpy of fusion obtained during the second heating scan, $\Delta H_{m}^{{}^{\circ}}$ is the enthalpy of fusion of completely crystalline PP ($\Delta H_{m}^{{}^{\circ}}$ = 209 J/g [32]), and *φ*_PP_ = 20% is the weight fraction of PP.

### 2.5. Rheological Characterization

The dynamic rheological characterization in the molten state of the native polymers and PS80/PP20 blends was accomplished with an imposed strain-controlled rheometer (RDA II, Rheometric Scientific Inc., New Castle, DE, USA), using parallel plate geometry (diameter 25 mm and gap 2.0 mm) in oscillatory motion. Before reaching the measurement position, the samples were melted in air atmosphere through a Pelletier element toward a controlled temperature of 200 °C and kept stable for 10 min, similar to the extrusion temperature. Prior to frequency sweep testing, a strain sweep test was performed to determine the region of linear viscoelastic response, and a strain amplitude was subsequently fixed at 10%. The frequency sweep tests were subsequently done in a frequency range from 0.01 to 100 rad s^−1^, while recording the storage modulus (*G*′), loss modulus (*G*″), and complex viscosity (η*). For conversion of complex viscosity into shear viscosity, the Cox–Merz rule │*η* * (ω)│ = │*η* (rad/s)│ was applied. Curve-fitting procedures for the polymer blends according to the modified Carreau–Yasuda model were applied to determine the zero-shear viscosity [46]. This model was chosen because, among the simpler models, it offers the greatest flexibility and, thus, the largest variation of the shape of the viscosity functions, making it very suitable for the description of viscosity functions in the terminal, as well as in the shear thinning regime.

## 3. Results and Discussion

### 3.1. Morphological Characterization

**Figure 1:**
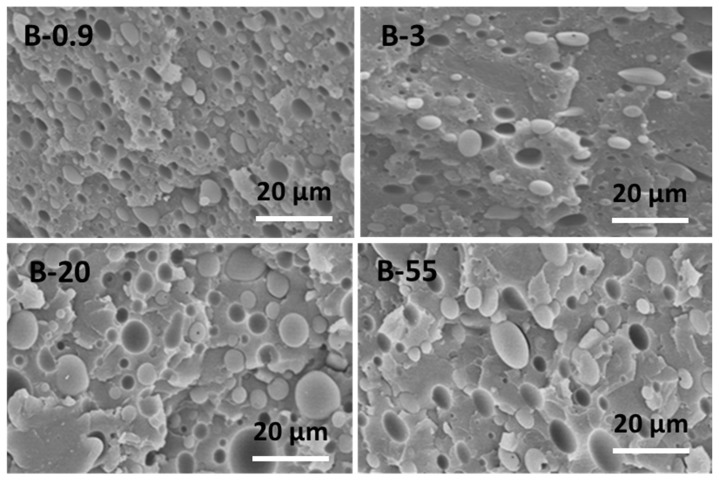
SEM micrographs of cryogenically fractured surfaces of PS80/PP20 blends with different viscosity ratios (*K*) in the transverse direction.

The morphology of PS80/PP20 blends with different viscosity ratios is shown in SEM micrographs in Figure 1, taken in a transverse direction to the flow. All blends exhibit a heterogeneous phase-separated morphology due to the immiscibility [47], with a dispersion of PP droplets as separated islands in the PS matrix. Some degree of plastic deformation on the fracture surfaces indicates that the fully brittle nature of native PS is covered in the blends. The observed cavities corresponding to the footprints of PP droplets indicate poor interfacial adhesion between PP and PS, caused by high interfacial tension between the blend constituents. Theoretically, a high viscosity ratio will result in large deformed droplets and a coarse morphology, whereas a low or matching viscosity ratio could result in fine fibrils and uniform morphology during melt-spinning [48]. There is visual evidence that interface detachment between both phases seems to occur most frequently for the fractured sample B0.9 (viscosity ratio, *K* > 1), where the viscosity of the PP particles is relatively higher than the PS matrix. Therefore, the shear deformation of the dispersed PP spheres is expected to be smaller than the PS matrix and may cause interface incompatibilities, resulting in gap formation between the blend phases. The gaps may form due to the relatively easy elongation of the matrix without deformation of the dispersed phase. On the contrary, the phase structure with dispersed PP spheres of low viscosity (*K* < 1) may present more easy flow and deformation than the more viscous PS matrix and create better compatibility. Therefore, the particle shape also tends to become more elliptical at the lower viscosity ratio due to deformation PP phase under shear. As such, the morphology of PS/PP blends can be tuned by a proper material selection based on their viscosity ratio, in agreement with earlier reports using the viscosity ratio of immiscible polymer blends to control the deformation of confined droplets and their orientation [49].

Depending on the volume fraction (*φ*_PS_, *φ*_PP_) and viscosity (*φ*_PS_, *φ*_PP_) of both phases, the morphology of immiscible PS/PP blends may change from intermediate phases into a co-continuous blend, according to the empirical relationship of Jordhamo in Equation (2) [50],

(2) $$\frac{\varphi_{PS}}{\varphi_{PP}}=\;\frac{\eta_{PS}}{\eta_{PP}}=\;\frac{1}{K}$$

or the corrected relationship between Chen and Su in Equation (3) [51]:

(3) $$\frac{\varphi_{PS}}{\varphi_{PP}}=1.2\;{\left(\frac{\eta_{PS}}{\eta_{PP}}\right)}^{0.3}=\;1.2\;{\left(\frac{1}{K}\right)}^{0.3}$$

From Equation (3), it is correctly concluded that the composition PS80/PP20 is far away from the co-continuity and phase inversion region for all viscosity conditions of PP in the present situation: Phase inversion would occur only at *K* = 0.02. The results from Equation (2) would imply a phase conversion at *K* = 0.25, which is not observed in the present case, as the latter simplified model is incorrect because it is limited to low shear conditions and does not correctly account for interfacial tension.

The results of statistical analysis of the blend morphology (Image-J software), i.e., shape of the dispersed PP phase, are presented in Table 3, including number average particle diameter (*d_n_*), weight-average particle diameter (*d_w_*), polydispersity (PDI = *d_w_*/*d_n_*), minimum size (*d_min_*), maximum size (*d_max_*), and calculated specific interfacial surface area of the blends (S_d_= 6 × *φ*_d_/*d*_p_ for a given volume fraction *φ*_d_ = 20% of the dispersed phase). Although the particle size distribution is relatively broad and a little fraction (<3%) of small particles (*d_min_* = 1 to 3 µm) remains existing for all blends, the mean sizes *d_n_* and *d_w_* of the dispersed PP particles significantly increase with higher viscosity ratio, *K*, while specific surface area of the dispersed PP particles progressively decrease with higher viscosity ratio, *K*. These observations are in contrast with earlier reports [3], where an increase in the viscosity ratio of PS80/PP20 due to lowering the viscosity of the matrix resulted in smaller particles. The increase in viscosity of the PS matrix also resulted in increased particle size for the compatibilized PS80/PP20 blends [3]. Otherwise, multiple theoretical and experimental results suggest that average particle size should decrease with increasing matrix viscosity, which seems to be valid when using a PS dispersed phase in PP matrix [52]. For the present conditions with variation in viscosity ratio due to increase in viscosity of the dispersed PP phase in PS matrix, the morphology clearly shows coarseness at a lower viscosity ratio, *K*. The strong tendency for coalescence of the minor phase (>20 wt.%) during melt-mixing has been noticed before [2]. The effects of break-up and coalescence of the particles seem to play an important role: it is evident that, (i) for blends with *K* >> 1, the low viscosity of the matrix relative to the PP serves as a lubricant preventing coalescence and the high viscosity of the PP prevents flow of the particles, while (ii) for blends with *K* << 1, the high viscosity of the matrix relative to the PP enhances the coalescence and low viscosity of the PP enhances flow of the particles. The higher shear stresses underflow induced by the matrix are consequently responsible for elongation of the dispersed PP particle into elliptical shape. As a result, sample B03 presents finest and most homogeneous microstructure. In contrast, the expected optimum for blend morphologies with finest dispersion at *K* ≈ 1, as determined in previous works [53,54], seems not fully valid in the present case. The benefits of having a highly viscous dispersed PP phase in a lower viscous PS matrix seem most favorable to develop small dispersed droplet sizes with good distributive and dispersive mixing.

**Figure 2:**
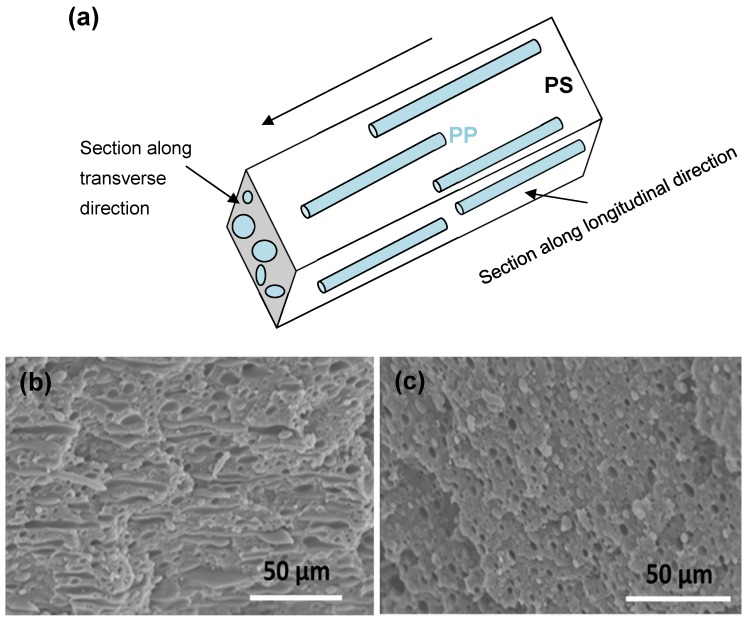
Detail of the microfibrillar morphology for dispersed PP in PS matrix developed by sample B0.9, in two directions relative to the flow direction in extrusion: (**a**) schematic of microfibrillar structure, (**b**) SEM image of section along longitudinal direction, and (**c**) SEM image of section along transverse direction.

The morphology of the dispersed PP phase was further analyzed by more detailed SEM images (Figure 2), which were taken in two sections of the material, along longitudinal and transversal direction, relative to the flow in extrusion processing. The SEM images show that the PP spontaneously forms a unique structure with an oriented microfibrillar shape along the flow direction, where the length of dispersed microfibers can reach up to 50 µm, and shape factor above 20. The microfibrils were only observed for sample B0.9 (*K* >> 1) and correspond to the formation of the finest homogeneous microstructure, while it did not occur at other viscosity ratios, *K*. These results are in agreement with those obtained by Miroshnikov and Williams and have important effects on the development of favorable mechanical properties [55]. Similar microfibrillar structures were observed for an elastic dispersed phase at high viscosity ratios and were ascribed to die-effects [56,57,58]. In contrast, the dispersed particles of PS within a PP matrix became more elongated in case of *K* < 1 [31]. The elongated interfaces were also reported for compatibilized PS80/PP20 blends and were attributed to plastic deformation and good interface adhesion [59]. In our previous studies, where the extrusion speed and cooling conditions were adapted for a PS90/PP10 blend, the formation of dispersed PP fibrillar structures also occurred under intense extrusion conditions at the very high shear rates [60,61]. It might, therefore, be expected that the introduction of shear stresses during extrusion causes the orientation of dispersed PP domains in the melt state and differences in relaxation times between both components depend on the viscosity. However, the deformation of a dispersed phase in an elongation flow field during extrusion seems to be insensitive to the viscosity ratio, as it was also observed during the mixing of a high-viscous matrix phase [62]. The formation of a continuous microphase of PP in PS80/PP20 blends has been denoted before [59], and was attributed to influences of the viscosity of the PP phase on the overall blend viscosity and uptake of the shear strain by the PP phase. Therefore, it seems more important to consider the melt viscosity of the entire blend (see later), as it is better related to the total mixing energy and internal shear forces: it is expected that a high viscosity of the blend will result in high internal shear stresses and dispersive forces causing deformation of the droplets without coalescence. The existence of elongation stresses was indeed demonstrated to be very efficient in break-up of droplets and consequent formation of a finely dispersed phase [63,64].

In relation with the present work, other studies on the morphology and deformation of dispersed phases in polymer blends can be compared. As one example, polyamide (PA6) drops in high-density polyethylene (HDPE) have been made in the case of ribbon extrusion [65]: This processing method is known to induce uniaxial orientation, which improves the properties of the extruded blend in the longitudinal direction rather than the transverse direction. Unfortunately, properties in the transverse direction are usually low. For other blending techniques, such as film-blowing, the polymer blend is biaxially oriented in the melt state, while it rapidly cools down, in order to freeze the oriented structure [66] The studies on the structure of polyethylene (PE) blown films, especially focused on the orientation and anisotropy of PE [67], where the lamellar structure of the film is the main parameter controlling anisotropy. In addition, molecular orientation imparted during film-blowing is known to have a major effect on the mechanical properties. Therefore, studying the film morphology is crucial to quantify the final properties of the films, especially for the orientation of dispersed domains in immiscible polymer blends [45]. In addition to cited reports, the present study also demonstrates the favorable development of microfibrils along longitudinal direction during extrusion of immiscible blends.

### 3.2. Melting and Crystallization Behavior

**Figure 3:**
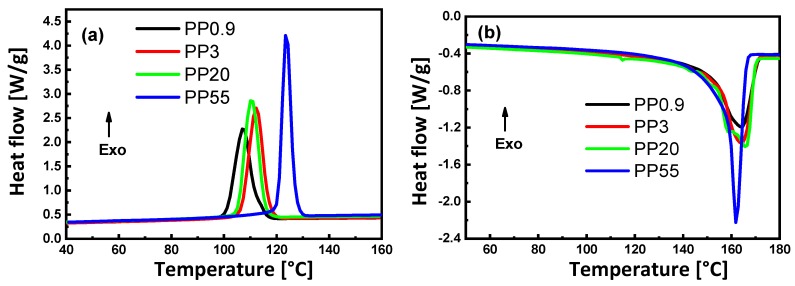
Thermal characterization for native polypropylene samples (PP0.9, PP3, PP20, and PP55) of different viscosities. (**a**) DSC thermograms showing the crystallization behavior, and (**b**) DSC thermograms showing the melting behavior.

**Figure 4:**
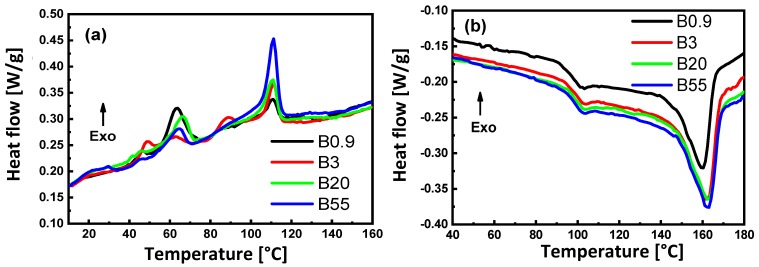
Thermal characterization of composites (B0.9, B3, B20, and B55) prepared with different viscosity ratios (4.32, 1.01, 0.15, and 0.12). (**a**) DSC thermograms showing the crystallization behavior, and (**b**) DSC thermograms showing the melting behavior.

The DSC analysis on melting and crystallization for native PP samples with different viscosities is presented in Figure 3. The DSC analysis of PS80/PP20 blends is presented in Figure 4, with a detail on the influences of viscosity ratio K on the degree of crystallinity (*X_c_*), melting temperature (*T_m_*) and crystallization temperature (*T_c_*) of PP, and glass transition temperature (*T_g_*) of the PS matrix. The thermal properties are summarized in Table 4 (native materials) and Table 5 (PS80/PP20 blends), respectively.

For native samples, the crystallization temperature (Figure 3a) and melting temperature (Figure 3b) are clearly affected by the viscosity of the PP grade. In a cooling cycle, the crystallization temperature for sample P55 with the lowest viscosity is much higher than the other PP grades, while the crystallization temperature further decreases gradually for the samples with higher viscosity. The effect of viscosity on crystallization temperature can be related to the need for a higher degree of undercooling for PP with higher molecular weight, as reflected in the lower MFI values because the lower degree of freedom of the long polymer chains (i.e., samples with low MFI) hinder the crystallization. The fractional crystallization of PP in a PS matrix obviously results in multiple crystallization peaks, which were not observed for the pure polymers. The latter likely result from confinement at the PS/PP interface [68]. Depending on the molecular structure of the polymer, however, the relation between MFI and crystallization temperature might not be linear or even random [69], and high MFI values may significantly enhance molecular mobility and crystallization above a certain threshold value due to lower molecular reputation effects. In a heating cycle, both the onset melting temperature and melting point increased at the higher MFI values probably as a result of larger crystallites formed. The reduction in melting temperature at the highest MFI (sample P55), however, suggests that the crystallization for low molecular material results in imperfect crystals: It is known that the low-molecular-weight fractions of isotactic PP seem to prevent chain folding and form a different crystal structure [70]. It was evidently noted that the degree of crystallinity of PP increased from 28.5 to 44.8% when the viscosity decreased, due to more easy crystallization of short length PP chains.

For PS80/PP20 blends, variations in the crystallization thermograms (Figure 4a and Table 4) are observed relative to the native materials and depend on the viscosity ratio. The crystallization thermograms display a complex behavior of the dispersed PP with multiple exotherms that are representative of a fractioned crystallization. This phenomenon was already reported by several authors [33,71] and was attributed to the size and shape of the dispersed phase, where each exotherm represents a population of dispersed domains whose crystallization was nucleated in different ways. In particular, the significant shifts in crystallization temperature *T_c_* of PS/PP blends were mainly observed for compositions below 50 wt.% PP [72]. The crystallization of dispersed PP domains induces a change in crystallization mechanism from heterogeneous nucleation (large domains) into homogeneous nucleation (small domains) with a consequent drop in *T_c_*. The crystallization exotherms are most prominent for the sample B0.9 and sample B55, in parallel with the most homogeneous morphology of either small or larger dispersed droplets, while the crystallization exotherms for other samples contain more intermediate fractions in parallel with the larger distribution of particle sizes. The crystallization for sample B0.9 is most intense at *T_c_* = 60 °C (lower than bulk PP0.9) and corresponds with homogeneous nucleation expected for the small dimensions and even dispersion of the PP phase. Although the *T_c_* for all blends is lower than the bulk PP grades, the peak shift T is different and more fractionated for sample B3 and sample B20, reflecting the more heterogeneous morphologies in comparison with sample B0.9, as described before. Different types of nucleation mechanisms may be active in different droplets of PP. The crystallization for sample B55 happens most intensely at *T_c_* = 111 °C and confirms the predominance of heterogeneous nucleation within the larger PP domains. In conclusion, the latter mechanisms support the fact that the large sizes of PP domains in samples with low *K* (sample B55) promote heterogeneous nucleation, and the smaller dimensions of the domains in samples with high *K* induce the homogeneous nucleation. The relative loss crystallinity parameter (*Y*) between the neat PP grades and the blends can be calculated by Equation (4):

(4) $$Y\left(\%\right)=\frac{\Delta H_{PP}-\Delta H_{blend}}{\Delta H_{PP}}\cdot 100\;$$

From Table 4, we can see that sample B0.9 has the lowest relative loss crystallinity, which relates to the specific fibrillar morphology and good homogeneity of a finely dispersed phase, in contrast with the heterogeneous morphologies and particle shapes of the other samples.

The melting temperature, *T_m_*, of the dispersed PP phase (Figure 4b and Table 4) systematically increases when the viscosity ratio, *K*, of the PS80/PP20 blend increases, while it remains lower than the bulk PP. The lowest *T_m_* in sample B0.9 is indicative of the presence of small crystallite sizes and supports the described homogeneous crystallization mechanism, while the steady increase in *T_m_* at lower viscosity ratio, *K*, is in line with the presence of larger crystals by heterogeneous nucleation. The reduction in *T_m_* for semi-crystalline polymers was frequently attributed to interactions between amorphous and crystalline phases. The reduction in *T_m_* was mainly for PS/PP blends at concentrations below 50 wt.% PP and attributed to the formation of small discrete crystalline domains [42]. Alternatively, a small reduction in *T_m_* was assigned to the interaction between the blend components [73], and it should presently be most pronounced in the case of sample B0.9, due to its finest and most homogeneous structure. In conclusion, the influence of the viscosity ratio on melting point reflects the quality of fine crystalline structures that form by homogeneous nucleation at *K* >> 1.

The glass transition temperature, *T_g_*, of the PS matrix (Figure 4b and Table 4) is higher for all PS80/PP20 blends compared to the PS bulk polymer, while it is not significantly affected by the viscosity ratio. The *T_g_* is in general mostly affected by the blend composition, while the upward shift is in the opposite direction than expected from the expected evolution of partially miscible blends toward and intermediate *T_g_* in between the lower *T_g_* of PP and the higher *T_g_* of PS. Therefore, constraint mobility of the amorphous PS matrix likely happens through the presence of dispersed PP droplets that act as barriers to the molecular relaxation processes of amorphous PS regions. However, no chemical interactions between the PS and PP phase could be detected, while implied physical constraints on molecular relaxation and mobility of the chain segments in the PS phase are more likely. For that reason, also the viscosity ratio, *K*, might not have an influence on *T_g_*, as all the dispersed droplets are in micrometer size ranges. Similarly, the mixing of inert glass beads as fillers (micrometer range) in polymers has equally demonstrated an increase in *T_g_* of the PS matrix, due to the formation of an immobilized interfacial layer and pinning of the molecular chains [74]. The increase in *T_g_* was previously also observed in PS/PP blends with PP as the matrix phase containing dispersed PS beads [5], where mechanisms were additionally attributed to variations in shrinkage between both phases, resulting in compressive stress on the PS phase.

### 3.3. Rheological Properties

**Figure 5:**
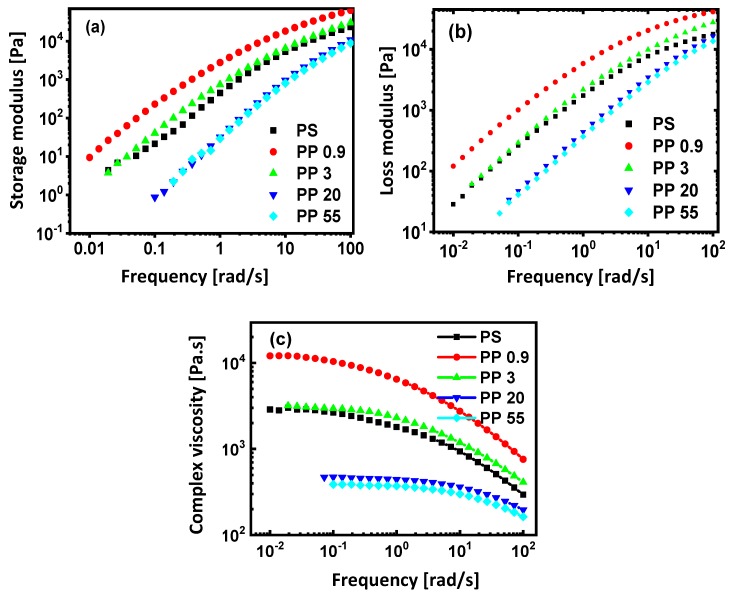
Evolution of (**a**) storage modulus, (**b**) loss modulus, and (**c**) complex viscosity for native polymer melts (PS and PP0.9, PP3, PP20, and PP55) as a function of frequency during oscillatory testing at 200 °C.

Dynamic oscillatory rheology measurements are widely used to characterize the viscoelasticity and structural changes in polymer composites [75,76]. The determination of the rheological properties of polymer blends in the molten state is crucial in order to gain a fundamental understanding of the processability of these materials [77]. The results of melt rheology testing for native polymers are compared in Figure 5, including the evolution of storage modulus (*G*′), loss modulus (*G*″), and complex viscosity (η*) as a function of frequency (ω) at 200 °C. The moduli *G*′ and *G*″ increase as a function of frequency with *G*″ > *G*′ over the majority of present testing range, as an indication for the predominant viscous response, and there is overlap in the range of tested PP grades with PS with similar values for PP3 and PS. A slight conversion into elastic behavior (G’ > G”) is only seen for the PP09 grade at above 60 s^−1^ and for the PP3 grade at above 100 s^−1^. This can be attributed to the hindrance of the molecular mobility at the higher frequencies and obviously occurs more frequently for PP grades with higher molecular weight (low MFI). Furthermore, the increase in MFI for different PP grades is in line with a steady decrease in moduli. Although the MFI values for PP20 and PP30 are significantly different, rheological properties remain comparable. The complex viscosity graph shows that both PP and PS have a characteristic Newtonian plateau at low frequencies and a decrease at higher frequencies characteristic for a pseudoplastic behavior.

The zero-shear viscosity was determined by fitting the experimental curves of shear rate sweep data through the modified Carreau–Yasuda model (see Supplementary Information Figure S1) for branched polymers [78,79] in Equation (5):

(5) η(γ˙)−η∞η0−η∞=(1+(λγ˙)a)(n−1)a 

with values for parameters *η*_0_ (zero-shear viscosity), λ (relaxation time constant), n (power-law exponent), and a (dimensionless parameter that describes the transition region between the zero-shear rate region and the power-law region) calculated in Table 6. The parameters *η*_0_, λ, and a were obtained by the best numerical fit of (Equation (5)) to the experimental data. The deviations between the fit and the measured data were compared, to check for the applicability of the fitting procedure [80]. In parallel with that, the values for viscosity ratio (*K*) were determined at different temperatures (see Supplementary Information Figure S2). The critical shear rate 1/λ corresponding to critical shear stress τ* = *η*_0_/λ represents a value where viscosity starts to deviate from Newtonian behavior and decreases by shear-thinning. The increase in critical shear rate and a decrease in critical shear stress for PP evidently relate to the decrease in molecular weight according to MFI values.

**Figure 6:**
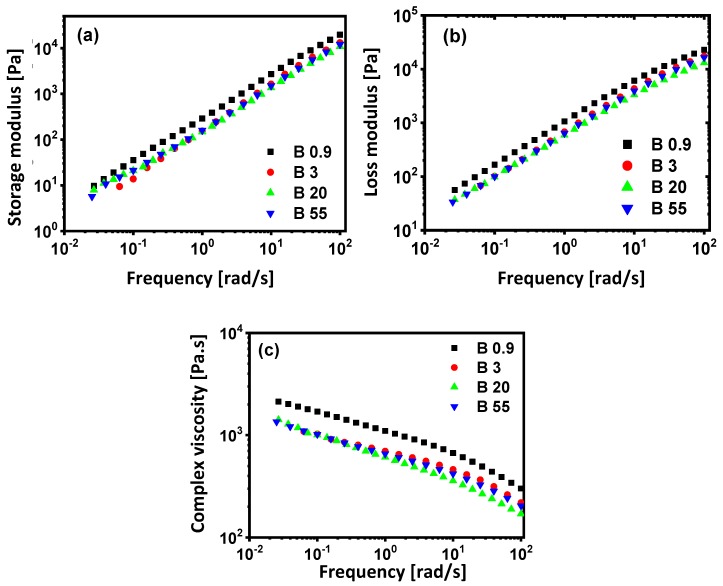
Evolution of (**a**) storage modulus, (**b**) loss modulus, and (**c**) complex viscosity for PS80/PP20 blends with different viscosity ratio (*K*) values as a function of frequency during oscillatory testing at 200 °C.

The rheological properties of molten blends, according to oscillatory testing at 200 °C, are illustrated in Figure 6. The addition of PP to PS matrix influences remarkably its rheological property over the whole frequency range. The storage moduli, *G*′, (Figure 6a) and loss moduli, *G*″, (Figure 6b) for PS80/PP20 blends are significantly smaller than those for neat PS and PP, with *G*″ > *G*′ over the full testing range showing viscoelastic behavior without transitions in B09 and B3, as observed for the native PP09 and PP3 grades, indicating lack of hindering molecular interactions between the phases. The presence of a shoulder in storage moduli, *G*′, in the low-frequency range is seen for blends B20 and B55: the more pronounced elastic modulus at low frequencies is typical of composite materials, and it is associated with the shape change of the dispersed phase in the matrix during oscillatory shear deformation [81,82]. In particular, the increase in *G*′ at low frequency for immiscible blends is specifically related to deformation and relaxation time of the deformed dispersed phase and becomes more evident for large particle sizes [83]. The latter has disappeared for the sample B09, with a perfect linear trend and highest values for *G*′ and *G*″ among all blends: The high elasticity modulus for those samples is in parallel with its fine and most homogeneously dispersed morphology, while the high dissipation is due to the large interface between matrix and fine microfibrils. These observations support the conclusions from the morphological analysis, providing a blend composition with fine morphology, best homogeneity, and interfacial compatibility at *K* > 1. Similar results in *G*′ and *G*″ were also reported while comparing un-compatibilized PS/PP blends to compatibilized PS/PP blends and upon reduction in particle size of the dispersed phase [84]. The complex viscosity (Figure 6c) for blends is significantly smaller than those for neat PS, possibly due to a slipping phenomenon between the phases [85]. However, the additive rule with a prospected increase in viscosity of PS upon addition of a more viscous PP component, in the case of sample B09, does not apply. In that case, the formation of a finely dispersed and homogeneous sample may be beneficial for a reduction in total blend viscosity relative to the pure PS matrix because of lubricating effects. Moreover, the addition of other PP grades with gradually lower viscosity does not relatively decrease the blend viscosity in samples B3, B20, and B55. The absence of a Newtonian plateau in viscosity plots at low frequencies is typically observed for all blends—in contrast with all native polymers—and it is an indication for effects of structural changes or molecular weight [86]. The significant increase in viscosity at low frequencies for samples B3, B20, and B55 relative to the native polymers P3, P20, and P55 and superposition of values for all blends suggest that hindrances in relaxation mechanisms of the matrix become predominant. The highest overall viscosity values for the blend in case of sample B09 seem to play a dominating role in the homogeneous and fine dispersion of the PP and formation of microfibrils. In conclusion, two cases can be distinguished for a given PS80/PP20 composition: (i) when *K* > 1, the rheological properties of the blend are dominated by the dispersed phase, and (ii) when *K* < 1, the rheological properties of the blend are dominated by the matrix.

Under conditions for extrusion in a mini-compounder, at 60 rpm and 200 °C, an internal shear rate of about 60 s^−1^ can be estimated from a proximate calculation. Under comparable conditions in frequency testing, the values for moduli *G*′ and *G*″, melt elasticity, and dissipation factors, elastic and dissipation ratio of the components, and loss factor tan *δ* = *G*″/*G*′ are calculated in Table 7, and they are comparable to the relative trend of viscosity ratio, *K*, between the samples, as given before. The high value of loss modulus *G*″ for sample B09 is in agreement with the morphology of smallest particles and best homogeneity, in parallel with general observations that the loss moduli for compatibilized systems are higher than those of un-compatibilized blends [87]. The possibility of interfacial slip for sample B09 (*K* < 1: “hard-in-soft” phase structure) may account for the dissipation of energy and higher loss modulus, *G*″. On the other hand, the low values of loss modulus, *G*″, for samples B20 and B55 (*K* < 1: “soft-in-hard” phase structure) indicate less interfacial interactions. Moreover, other blends with a homogeneous distribution of well-formed spherical particles as a dispersed phase in a continuous matrix showed an increase in loss moduli [88]. The loss factor is related to internal damping and is generally used as a measure to quantify miscibility, intermolecular interactions, interface features, and morphology. The lowest value of loss factor for the blend B09 among other blends indicates that there are small energy losses during mixing, and lowest energy input is required to create the most homogeneous blend with microfibrils at *K* > 1.

**Figure 7:**
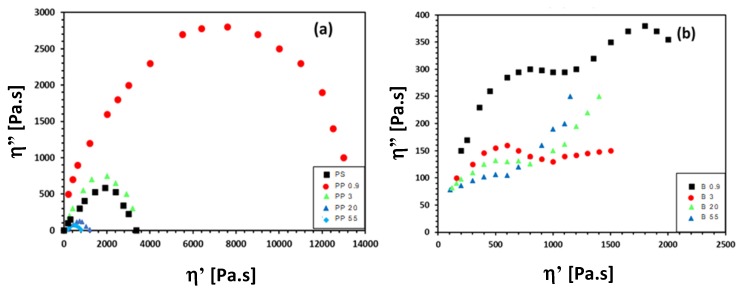
Cole–Cole plots illustrating relaxation behavior for (**a**) native PP and PS grades, (**b**) PS80/PP20 blends with different viscosity ratio (*K*).

A relation between the different morphologies and relaxation times of the polymer blends can be illustrated from the Cole–Cole plots (Figure 7), representing the imaginary part of viscosity (*η*″ = *G*′/ω) as a function of the real part of viscosity or dynamic viscosity (*η*′ = *G*″/ω). The plot describes the viscoelastic properties of a material with a relatively broad distribution of relaxation times, forming either a semicircle in the case of a single relaxation (e.g., homogeneous material), or a deviating profile with multiple semicircles and tails for a composition with multiple relaxation times (e.g., filled systems, composites, or blends). For the native materials (Figure 7a), the relaxation process regularly follows a semicircle profile as a characteristic for its homogeneous composition. The larger radius of the plot corresponds to longer relaxation times: the relaxation time for the various PP grades gradually decreases with higher MFI in parallel with the expected effect of lowering the molecular weights (higher MFI) inducing more easy relaxation based on reduced molecular entanglements. Due to the selected range in MFI, the relaxation of the PP09 grade is a significant order of magnitude above the other PP grades. The relaxation of the PS grade is situated around the properties of the PP3 in parallel with the reported values of the zero-shear viscosity. The PS80/PP20 blends (Figure 7b) interestingly show a relaxation profile with occurrence of multiple semicircle profiles or tails at the outer end, as a result of the immiscibility and presence of a multiphase structure for all compositions. The first relaxation mechanism corresponding to high frequencies is attributed to the main component (i.e., PS matrix), followed by a second relaxation process at low frequencies due to the deformation of the dispersed phase. The latter obviously creates two interfering semicircles for sample B09, with the second one corresponding to the higher relaxation times, as the microfibrillar structure can be correlated to a significant delay in the relaxation process. The observed relaxation process indicates high shape elasticity and interfacial tension for the microfibrillar sample B09. In particular, the very fine structures of thin droplets and good interfacial cohesion between phases cause a delay in the relaxation times, as also observed as separate circular profiles for compatibilized blends. The other blend compositions typically show the formation of a tail at the outer end, which occurs more readily for samples B3, B20, and B55 in parallel with its morphology of bigger PP droplets and microstructural heterogeneity. The appearance of a sudden linear increase indicates higher immiscibility and poor dispersion between the phases. In conclusion, the presented analysis confirms that the observed morphology can be explained in parallel with the relaxation properties of the blends, where the fibrillar structure causes an intimate structure with retarded relaxation in contrast with the inhomogeneous droplet structure.

## 4. Conclusions

The competition in developing a microfibrillar structure or individual droplets of the dispersed PP phase in a PS80/PP20 blend was analyzed as a function of the viscosity properties of the dispersed phase by keeping the matrix properties constant (viscosity ratio *K*): the cryo-fractured blend shows a microfibrillar morphology formed by the dispersed phase for high *K* >> 1, while droplets with gradually increasing sizes (diameter) were formed at lower *K* ≈ 1 or *K* << 1. The variations in morphology are further explained by thermo-analytical properties and melt-rheological behavior. The crystallization for blends with a fine microfibrillar structure is most intense at *T_c_* = 60 °C (lower than bulk PP0.9) and corresponds with a homogeneous nucleation expected for the small dimensions and even dispersion of the PP phase, whereas the blends with droplets of various sizes show a broad distribution in crystallization temperatures in parallel with different nucleation mechanisms. The lowering in the melting temperature of microfibrillar PP is indicative of the formation of small crystallite sizes and can also be assigned to the greater interaction between the blend components, in contrast with the droplet morphologies. The rheological properties show a perfect linear trend and highest values for *G*′ and *G*″ for microfibrillar blends, indicating good homogeneity and interaction between the blend components. In particular, the highly complex viscosity of the microfibrillar blend seems to play a dominating role in the homogeneous and fine dispersion of the PP and formation of microfibrils. The particular microfibrillar morphology is most effectively expressed in the relaxation diagrams, indicating multiple relaxation processes of the matrix and a retarded relaxation of the dispersed phase for the fine homogeneous microfibrillar blends, in contrast with the blends having coarse droplet structures showing instable relaxation properties.

## Figures and Tables

**Table 1:** Sample codes and characteristics of polystyrene (PS) used as a matrix phase and isotactic polypropylene (iPP) used as the dispersed phase.

Sample Grade	Sample Code	Density at 23 °C ^1^ (g/cm^3^)	Melt Flow Index at 200 °C ^1^ (g/10 min)	Zero-Shear Viscosity ^2^ *η*_0_ (Pa·s) at 200 °C	Supplier
PS	PS	1.050	/	3150	BASF S.A.
100GA01	PP0.9	0.923	0.9	13349	Ineos
ISPLEN PP-040G1E	PP3	0.905	3.0	3199	Repsol YPF
ISPLEN PP080G2M	PP20	0.905	20.0	466	Repsol YPF
ISPLEN PP099K2M	PP55	0.905	55.0	389	Repsol YPF

^1^ According to supplier data; ^2^ from the fit of the Carreau–Yasuda model (see later in Equation (5)) to the zero shear viscosity data.

**Table 2:** Sample codes of prepared PS80/PP20 blends with different viscosity ratio *K* = ($\eta_{0,\mathrm{PP}}/\eta_{0,\mathrm{PS}}).$

Blend	PS + PP0.9 (80/20)	PS + PP3 (80/20)	PS + PP20 (80/20)	PS + PP55 (80/20)
Code	B0.9	B3	B20	B55
*K*^1^ at 200 °C	4.23	1.01	0.15	0.12

^1^ From the fit of the Carreau–Yasuda model (see later in Equation (5)) to the zero shear viscosity data.

**Table 3:** Statistical analysis of particle size distribution of dispersed PP phase in PS80/PP20 blends with different viscosity ratios (*K*).

Blend	*d*_n_ (µm)	*d*_w_ (µm)	PDI	*d_min_* (µm)	*d_max_* (µm)	S_d_
*B09*	2.80	3.07	1.10	1.0	6.0	0.43
*B3*	6.38	7.04	1.10	2.5	12.3	0.19
*B20*	9.03	9.82	1.09	2.5	15.5	0.13
*B50*	10.3	11.1	1.098	3.0	17.0	0.12

**Table 4:** Thermal characteristics for neat PPs and PS polymers.

Polymer	*T_c_* (°C)	*T_g_* (°C)	*T_m_*_(Onset)_ (°C)	*T_m_* (°C)	$X_{c}\;(\%)$
PS	-	95.5	-	-	-
PP0.9	110.5	N/D ^1^	153.9	168.9	28.5
PP3	112.3	N/D ^1^	154.6	168.7	35.1
PP20	112.8	N/D ^1^	157.9	170.9	34.1
PP55	124.6	N/D ^1^	149.9	165.1	44.8

^1^ N/D = not determined.

**Table 5:** Thermal characteristics for the PP/PS blends.

Blend	*T_c_* (°C)			*T_g_* (°C)	*T_m_* (_Onset_) (°C)	*T_m_* (°C)	$X_{c}\;(\%)$	Relative Loss Crystallinity (%)
	Peak A	Peak B	Peak C					
*B0.9*	110.0	63.5	45.0	98.7	145.5	160.0	24.7	13.33
*B3.0*	111.0	88.0	48.4	98.6	149.5	161.8	25.8	26.49
*B20*	110.7	66.1	42.0	98.0	149.6	162.8	28.2	17.30
*B55*	111.1	64.2	-	99.9	151.8	163.1	19.3	56.91

**Table 6:** Rheological parameters by the experimental fit of the modified Carreau–Yasuda model for the native polymers at 200 °C.

Polymer	$\eta_{0}\;(\mathrm{Pa}\cdot s)$	λ (s)	*n*	*A*	1/*λ* (s^−1^)	τ* (Pa)
PS	3150 ± 86	0.517 ± 0.039	0.534 ± 0.075	0.457 ± 0.097	1.93	6092
PP0.9	13,349 ± 186	1.020 ± 0.291	0.564 ± 0.037	0.419 ± 0.039	0.98	13,087
PP3	3199 ± 19	0.508 ± 0.079	0.736 ± 0.033	0.495 ± 0.023	1.97	6297
PP20	466 ± 3	0.140 ± 0.043	0.699 ± 0.034	0.975 ± 0.120	7.14	3329
PP55	389 ± 2	0.114 ± 0.029	0.670 ± 0.031	0.933 ± 0.085	8.77	3412

**Table 7:** Elasticity and dissipation factors of native polymers and blends under extrusion conditions (60 s^−1^, 200 °C).

	*G*′ (Pa)	Melt Elasticity (Pa·s)	Melt-Elasticity Factor	*G*″ (Pa)	Melt Dissipation (Pa·s)	Melt Dissipation Factor	Loss Factor tan *δ*
PS	1.5 × 10^4^	250	-	1.6 × 10^4^	266	-	
PP0.9	5.1 × 10^4^	850	-	3.2 × 10^4^	530	-	0.62
PP3	2.0 × 10^4^	333	-	2.3 × 10^4^	380	-	1.15
PP20	0.8 × 10^4^	133	-	1.2 × 10^4^	200	-	1.50
PP55	0.5 × 10^4^	83	-	0.9 × 10^4^	150	-	1.80
B0.9	1.4 × 10^4^	233	3.4	1.8 × 10^4^	300	0.56	1.29
B3	0.9 × 10^4^	150	1.3	1.4 × 10^4^	233	0.44	1.56
B20	0.8 × 10^4^	133	0.53	1.3 × 10^4^	216	0.40	1.63
B55	0.7 × 10^4^	116	0.33	1.2 × 10^4^	200	0.36	1.72

## Supplementary Materials

The following are available online at https://www.mdpi.com/1996-1944/13/4/926/s1, Figure S1: Curve-fitting according to Carreau–Yasuda model for PP3, Figure S2: Evolution of complex viscosity for native polymer melts as a function of frequency during oscillatory testing at different temperatures of 160, 180, 200, and 220 °C for two materials, (a) PS, (b) PP3. Table S1: Temperature dependency of viscosity, *η*, and viscosity ratio, *K*.
