# Supplementary material for: Morphology, Rheology and Crystallization in Relation to the Viscosity Ratio of Polystyrene/Polypropylene Polymer Blends
Source: Materials (Basel). 2020 Feb 19;13(4):926. doi: 10.3390/ma13040926 (PMC7078875; doi:10.3390/ma13040926)
Supplement: Supplementary file 1 [file materials-13-00926-s001.pdf]

materials

Supplementary Materials

Morphology, Rheology and Crystallization in Relation to the Viscosity Ratio of Polystyrene/Polypropylene Polymer Blends

Salim Hammani [1] and Nadji Moulai-Mostefa [2], Pieter Samyn [3], Mikhael Bechelany [4,*], Alain Dufresne [5], Ahmed Barhoum [6,*]

[1] Laboratoire de Chimie Physique Moléculaire et Macromléculaire, Faculté de Science, Université de Blida109000 Blida, Algeria; hammani71@yahoo.fr
[2] Laboratoire Matériaux et Environnement, Faculté des Sciences & Technologie, Université de Médéa, Ain D'Heb, 26001 Medea, Algeria; moulai_nadji@yahoo.fr
[3] Applied and Analytical Chemistry, Institute for Materials Research (IMO-IMOMEC), Hasselt University, 3590 Diepenbeek, Belgium, pieter.samyn@uhasselt.be
[4] Institut Européen des Membranes IEM, UMR 5635, Univ Montpellier, ENSCM, CNRS, 34095 Montpellier Cedex 5, France.
[5] Univ. Grenoble Alpes, CNRS, Grenoble INP, LGP2, 38000 Grenoble, France; alain.dufresne@pagora.grenoble-inp.fr
[6] Chemistry Department, Faculty of Science, Helwan University, Ain Helwan, 11795 Cairo, Egypt.
* Correspondence: mikhael.bechelany@umontpellier.fr (M.B.); ahmed.barhoum@science.helwan.edu.eg (A.B.)

Received: 3 January 2020; Accepted: 14 February 2020; Published: February 2020

The zero-shear viscosity was determined by fitting the experimental curves of shear rate sweep data through the modified Carreau–Yasuda model. An example of curve fitting is shown in Figure S1 for the PP3 polymer. Others were determined in analogy, from the data shown in Figure 5.

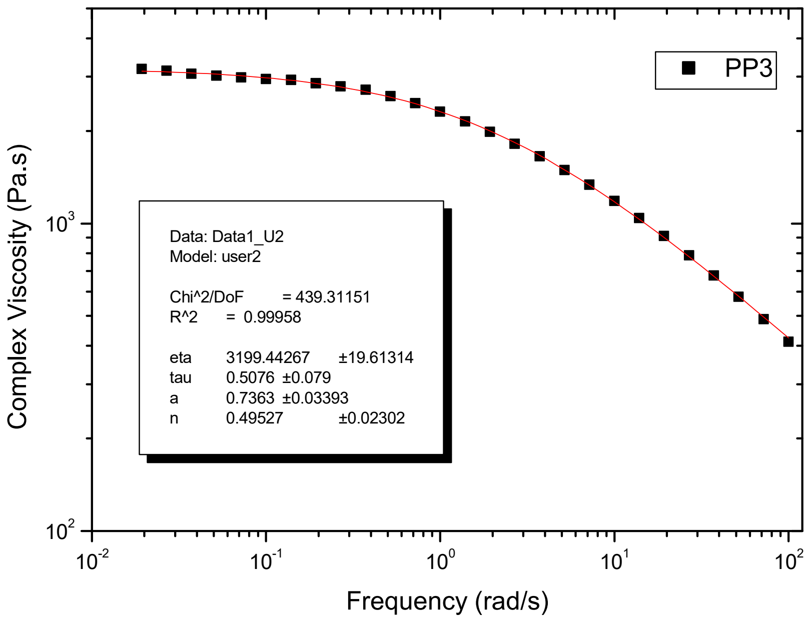

Figure S1. Curve-fitting according to Carreau–Yasuda model for PP3.

The values for the viscosity, *h,* and viscosity ratio, *K,* were determined at different temperatures, as summarized in Table S1. The values for zero-shear viscosity were determined by fitting the

experimental curves of shear rate sweep data, as shown in Figure S2, for two materials, through the modified Carreau–Yasuda model, as explained before.

Table S1. Temperature dependency of viscosity, [illegible], and viscosity ratio, *K*.

Temperature (°C)	$\eta_{0,PP}$ (Pa.s)	$\eta_{0,PS}$ (Pa.s)	K = $\eta_{0,PP}/\eta_{0,PS}$
160	7.10^3	6.10^4	0.1
180	5.10^3	1.10^4	0.5
200	$3.5.10^3$	3.10^3	1.1
220	3.10^3	$1.2.10^3$	2.5

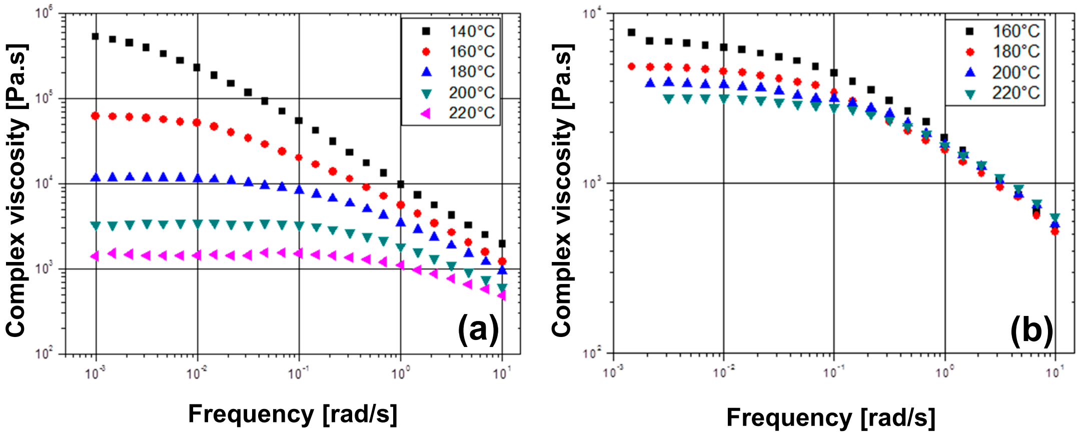

Figure S2. Evolution of complex viscosity for native polymer melts as a function of frequency during oscillatory testing at different temperatures of 160, 180, 200, and 220 °C for two materials, (**a**) PS, (**b**) PP3.
